# Validated green spectrophotometric kinetic method for determination of Clindamycin Hydrochloride in capsules

**DOI:** 10.1186/s13065-021-00755-0

**Published:** 2021-04-30

**Authors:** Shaza Affas, Amir Alhaj Sakur

**Affiliations:** 1 Analytical and Food Chemistry Department, Faculty of Pharmacy, Ebla Private University, Aleppo, Syria; 2 Analytical and Food Chemistry Department, Faculty of Pharmacy, University of Aleppo, Aleppo, Syria

**Keywords:** Clindamycin Hydrochloride, Spectrophotometry, Kinetic, Potassium Iodide, Potassium Iodate, Tri Iodide

## Abstract

**Background:**

simple, sensitive, free of organic solvents, kinetic spectrophotometric method has been developed for the determination of Clindamycin Hydrochloride, both in pure form and Capsules. Method is based on reaction of Clindamycin with potassium iodide and potassium iodate in an aqueous medium at (25 ± 2 °C) to produce yellow-coloured tri iodide ions (I_3_^−^). The reaction is followed spectrophotometrically by measuring the absorbance at wavelength 350 nm during 40 min.

**Results:**

the effects of analytical parameters on reported kinetic methods were investigated. Under the optimized conditions, the initial rate and fixed time (at 10 min) methods were used for constructing the calibration graphs. The graphs were linear in concentration ranges 1–20 μg ml^−1^ with limit of detection of 0.12 and 0.22 μg ml^−1^for the initial rate and fixed time methods, respectively. The results were satisfactory and the analytical performance for both methods was validated**.**

**Conclusion:**

The proposed methods have been applied to determine the components in capsules with an average recovery of 98.25–102.00% and the results are in good agreement with those found by the reference method.

## Introduction

**Fig. 1:**
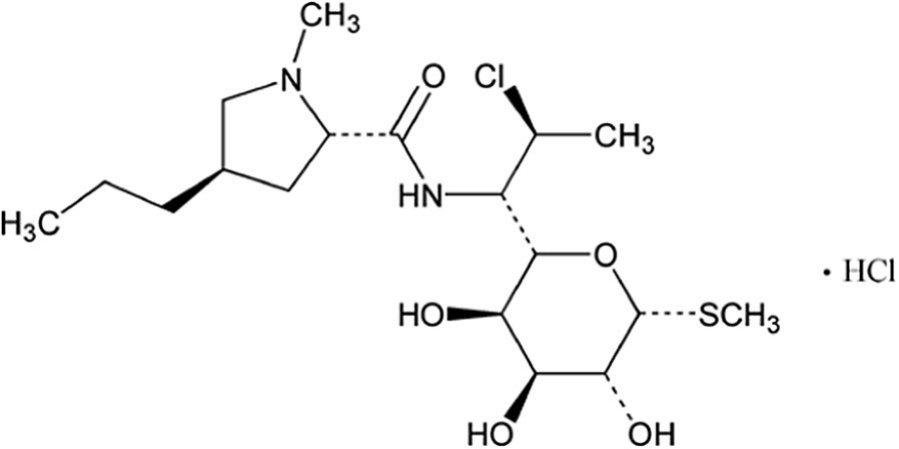
Clindamycin Hydrochloride

Clindamycin (CLN), (methyl-7-chloro-6,7,8-trideoxy-6-{[4R)-1-methyl-4-propyl-L-prolyl]amino}-1-thio-L-threo-3-Dgalacto-octopyranoside), is a semi-synthetic analog of lincomycin [1]. (Fig. 1).

By binding to the 50S subunit of the ribosome, Clindamycin inhibits the synthesis of bacterial proteins. It is active against Gram-positive aerobic, anaerobic and some Gram-negative aerobic bacteria. [1]

It works similar to a bacteriostatic antibiotic. Common clinical conditions in which they have included the infections of gynaecology, gingiva, respiratory tract, skin, soft tissue, and intra-abdominal infections. Also, Clindamycin is used in pneumonia caused by Pneumocystis, toxoplasmosis, malaria, babesiosis, and acne[2]. Clindamycin is existing in a wide variety of prescription formulations that can be given orally or dermally [2].

There are a variety of analytical techniques to determine Clindamycin in various pharmaceutical formulations, as well as in biological samples.

The United States Pharmacopoeia (USP) suggested the technique of liquid chromatography for the Clindamycin assay [3]. High-performance liquid chromatography (HPLC) is commonly used [4–9], potentiometric determination [10] capillary electrophoresis [11], micellar chromatography [12], chemiluminescence methods [13], voltammetry [14–16] and spectrophotometric determination that dependent on color forming [17, 18].

Previous publications reported spectrometric determination of CLN based on the method oxidation of a sulfur atom in acidic medium. Then the absorbance was measured at 520 nm [17]. Other spectrophotometric method includes the formation of ion-pair complex between CLN and rose bengal in faintly basic medium (pH 7.5). The produced colour is measured at 555 nm [18]

The proposed UV–Vis spectrophotometric method allows evaluating the content of CLN in capsules.

There is no research about the determination of CLN based on kinetic methods.

Kinetic methods have benefits such as reduction of interference with excipients, therefore there is a need for a Kinetic Method to determine CLN.

The purpose of this thesis was to report new, simple and accurate kinetic spectrophotometric methods for the determination of CLN.HCl as raw materials and in capsules without interaction with other ingredients in their formulations.

The proposed method is economical and low-cost since it uses inorganic reagents.

## Apparatuses

UV–Visible spectrophotometer (JASCO, model V650, Japan).

1.00 cm quartz cells.

An ultrasonic processor (Powersonic, model 405, Korea) was used to sonicate the sample solutions. Adjustable micropipettes covering a volume range from 2 to 2000 μL (ISO-LAB, Germany), used for the preparation of the experimental solutions.

Analytical balance (Sartorius, model 2474, Germany).

## Materials

Pharmaceutical grade Clindamycin HCl (99%) were received from XUHUANG, CHINA.

Potassium iodide and potassium iodate (Panreac, Germany).

All chemicals used were analytical grade.

## Standard solutions

By dissolving 25 mg of CLN.HCl in 25 ml of double-distilled water, a standard solution (1 mg ml^−1^) of CLN.HCl was prepared. Appropriate amounts of KI and KIO3 were dissolved in water for preparing solutions with a concentration of 0.3 M and 0.2 M, respectively.

The solutions were stable for a time of 2 days when stored at (5 °C).

## Approaches

### General Procedure for Kinetic Study

Increasing amounts of CLN.HCl standard solution (1 mg.ml^−1^) have been moved to 10 ml volumetric flasks containing 3 ml KI (0.3 M) and 1 ml KIO3 (0.2 M).

The volume was completed to the mark with water.

The absorbance of the prepared solutions was measured at different times 0, 5, 10,15, 20, 25, 30, 35 and 40 min. The λ_max_ was 350 nm.

At room temperature (25 ± 2 °C), the measurements were carried out.

## Procedure for calibration

### Initial rate method

Initial reaction rates were measured by calculating the slopes of the initial tangent to the absorbance time curves.

To reach concentrations between (0.5–30) μg ml^−1^ of CLN.HCl, aliquots of the CLN.HCl research solution is pipetted into 10 ml regular flasks.

In each flask, 3 ml of KI (0.3 M) and 1 ml of KIO_3_ (0.2 M) were added. Then they were diluted with distilled water to the mark.

The contents of each flask's mixture were well mixed.

The growth in absorbance was listed as a function of time at 350 nm. From the slope of the tangent to the absorbance-time curve, the initial reaction rate (n) at different concentrations was obtained.

### Fixed time method

In this method, the absorption of yellow-coloured solutions containing deferent quantities of the drug was measured at a certain fixed time, 10 min, as defined above for the initial rate method.

### Calibration structure

To construct a calibration curve, the absorbance information of kinetic tracks at 0 min and 40 min are used. The average relative responses of 5 repeats were estimated. Absorbance that comes between 98 to 102% of the average relative response is only included in the calibration curve construction For each drug, the limits of Beer's law, slope, intercept, coefficient of correlation and regression equation are brief in Table 1.

**Table 1:** The regression equation and Linear range for CLN.HCl at a fixed time and 25 ° C

Time (min)	Regression equation	R^2^	Linear range
300	A = 0.0883C − 0.0098	0.9996	1–20 mg/L
600	A = 0.0996C − 0.0064	0.9999	1–20 mg/L
900	A = 0.1053C − 0.0023	0.9998	1–20 mg/L
1200	A = 0.1078C + 0.0069	0.9998	1–20 mg/L
1500	A = 0.1103C + 0.0042	0.9997	1–20 mg/L
1800	A = 0.1139C − 0.0026	0.9999	1–20 mg/L
2100	A = 0.1146C + 0.0126	0.9997	1–20 mg/L
2400	A = 0.119C + 0.0101	0.9994	1–20 mg/L

*A* absorbance, *C* concentration

### Method validation

In terms of accuracy, precision and detection limits, the innovative method for estimating the drug has been validated.

Absorbance-time curves were drawn, and the substance recovery was measured using the initial rate and fixed time method.

The analyses have been replicated at least 5 times to assess the precision, and accuracy is measured in terms of percent recovery and percent RSD. The precision and accuracy of the procedures are demonstrated by strong percent recovery; the RSD was less than 2.

T-test and F-test values were both calculated using a reference method.

The t-test and F-test values are in the acceptable range, reflecting the methods' high accuracy and precision (Table 2).

**Table 2:** Parameters of the fixed time method (600 s)

Parameters	CLN
	Fixed time method
Linear rang μg.ml^−1^	1–20
ɛ l mol^−1^ cm^−1^	45.135 × 10^3^
Detection limit μg.ml^−1^	0.12
Limit of quantification μg ml^−1^	0.39
Regression equation	*($\mathrm{A }=\mathrm{ m C }+\mathrm{ b})$
	m = 0.099
	b = 0.006
Correlation coefficient	0.9999

^*^ Concerning A = mC + b, C means the concentration (µg.ml^−1^)

*A* absorbance

### Method selectivity and ruggedness

The excipients of each product were added to the pure drug sample. The recovery experimentations were performed. Ruggedness is method resistance for a minor variation such as apparatus and expert, or both. 3 different instruments and 2 analysts were reported to test the robustness of the absorption method results. No important changes were detected either by apparatus or analyst alteration.

### Procedure for pharmaceutical formulations

The content of twenty separate capsules was weighed up. An accurately weighed quantity of the powder equivalent to 50 mg of CLN.HCl was moved into a volumetric flask and dissolve in 50 ml of water. The content was sonicated for 10 min. A portion of this solution was centrifuged for 15 min at 5000 rpm, a suitable volume of the supernatant was moved into a 10 ml volumetric flask and procedure were continued to use for the analysis of CLN.HCl by the proposed spectrophotometric method.

## Results

**Fig. 2:**
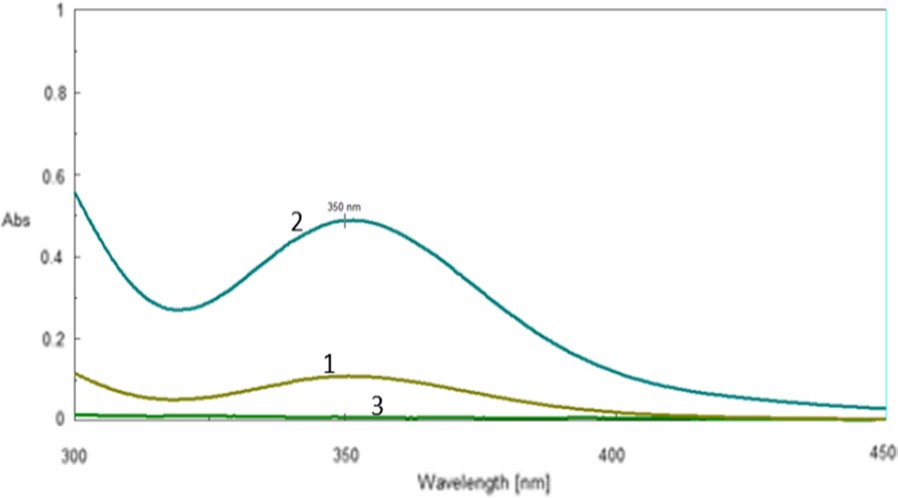
(1) Blank (I + KI) against distilled water, (2) CLN.HCl (5 μg ml^−1^) + 3 ml of 0.30 M KI + 2.0 ml of 0.1 M KIO_3_ against Blank, (3) CLN against water

Iodide ions transform to free iodine in an aqueous medium which has acidic properties, the acidity comes from HCl. Free iodine reacts with an excess of iodide ions and produces a yellow complex. The complex consists of iodide (I_3_^−^) (Fig. 2) [19, 20].

$${{\text{IO}}}_{{3}}^{ - } + {8} {{\text{I}}}^{ - } + {6} {{\text{H}}}^{ + } \to {3} {{\text{I}}}_{{3}}^{ - } + {3} {{\text{H}}}_{{2}} {{\text{O}}}$$

Optimum conditions for interaction.

The ideal conditions for the improvement of the method were recognized by changing the parameters one at a time and keeping the others fixed and observing the influence produced on the absorbance of the coloured products. [19]

A volume of 3 ml of 0.3 M KI, 1 ml of 0.1 M KIO_3_ were found to be optimum for maximum colour advance for an estimate of CLN.HCl.

At the mentioned parameters, the results indicate that the absorbance value at 25 ◦C was maximum.

### Calibration graphs

The colour that appears in the solutions when the drug interacts with the reagents increases over time. This phenomenon can be used in a kinetic study to determine clindamycin.

The methods of the initial rate, rate constant, fixed absorbance and fixed time were studied.

Fixed time method and initial rate method were adopted due to accuracy, precision and linear range.

Therefore, the two methods were used for constructing the calibration and to determine CLN.HCl.

**Fig. 3:**
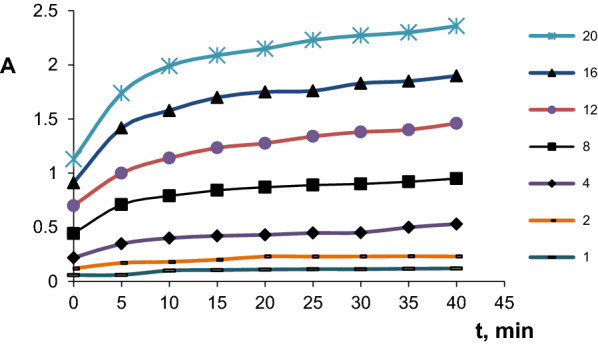
Absorbance—time curve for CLN.HCL + reagent (KI + KIO_3_), Concentrations of CLN.HCL:: (1)1 ppm; (2)2 ppm; (3)4 ppm;(4)8 ppm; (5)12 ppm, (5)16 ppm, (6) 20 ppm

Absorption changes were studied at λ_max_ (360 nm), for CLN-reagent solutions versus time (Other conditions were stable), as shown in Fig. 3—the concentration range of (1–20)mg/L-

### Initial rate method

The initial reaction rate was determined from the slope of the tangent to the absorption time curve at different concentrations. By plotting the logarithm of the initial reaction rate against the logarithm of the molar CLN.HCl concentration, the calibration graph was constructed.

The rate data of reaction would follow a pseudo order rate constant and submitted the following rate equation: [19]

$$\upsilon = \frac{{\Delta {\text{C}}}}{{\Delta {\text{t}}}} = \frac{{\Delta {\text{A}}}}{{\Delta {\text{t}}}} = {\text{K}}.{\text{C}}^{{\text{n}}}$$

where:

ν: reaction rate, A: absorbance., ∆A = At_2_ − At_1_, t is the measuring time, ∆t = t2 − t1, K is the pseudo order rate constant, c is the concentration of (CLI) mol/L and n is the order of the reaction. [21]

**Fig. 4:**
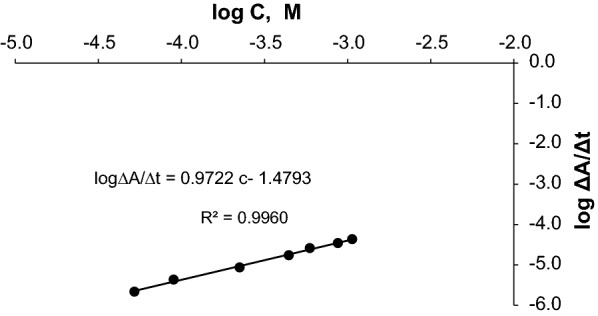
logarithm **(ΔA/Δt)** versus the logarithm of (**C**_**CLN**_) curve.

A calibration curve was constructed by plotting the logarithm of the initial rate of reaction (log ν) against the logarithm of (CLN.HCl) concentration (log c), which showed a linear relationship over the concentration range of (1–20) mg/L (Fig. 4). [22]

The equation of the curve can be written as follows:

$${\text{log v }} = {\text{ log }}\Delta {\text{A}}/\Delta {\text{t }} = {\text{ log k}}^{\prime} + {\text{ n logc}}$$

$${\text{log v }} = {\text{ Log }}\Delta {\text{A}}/\Delta {\text{t }} = \, 0.{\text{9722 log c }} - { 1}.{4793}$$

The reaction is the first order (n = 0.9722 = 1) regarding to Clindamycin concentration, k' = 30.15 s^−1^.

### Rate constant method

The logarithm of the absorbance contrasted with time for each concentration of CLN.HCL which investigated over the concentration range of 1–20 mg/L was estimated.

**Fig. 5:**
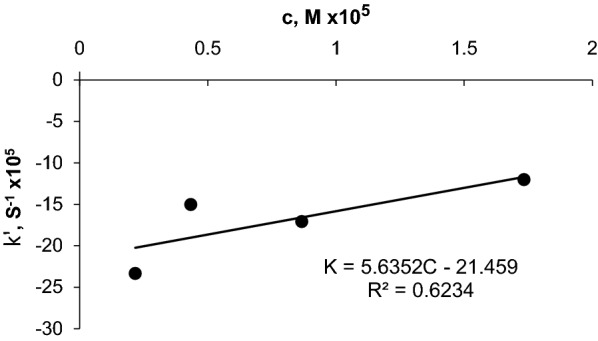
Rate constant method calibration curve for CLN.HCl

In the range of 1–8 mg/L (1.570 × 10^−4^_ 2.355 × 10^–4^) M, graphs of log absorbance against time were plotted for CLN.HCl concentration. The pseudo order rate constant (k') corresponding to CLN.HCl concentrations are determined from the slopes multiplied by-2,303[21]. The results are shown in (Fig. 5).

c against k' equation: $${\text{y}} = \, 5.6352{\text{x }} - \, 21.459 \, \left( {{\text{ R}} \, = \, 0.6234} \right)$$.

### Fixed absorbance method

The absorbance was fixed at the value 1.2, then the time required to reach this value was calculated in seconds for several concentrations of (CLN.HCl) in the range 10–20 mg/L.

It was found that the chosen value intersects with the curves of the absorbance change as a function of time.

**Fig. 6:**
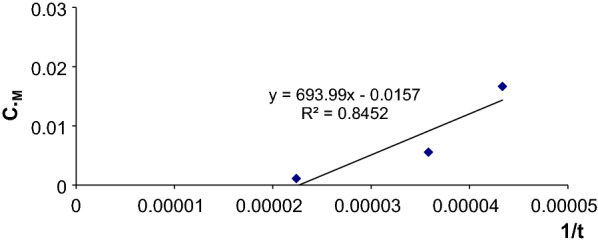
Graph for determining CLN.HCl by fixed absorbance method

Then the graph representing the changes in the time value of 1/t versus the concentration of ClN.HCl was plotted (Fig. 6).

Subsequently, the equation of the calibration graph was found:

$${1}/{\text{t }} = { 693}.{\text{99C }} - \, 0.0{157}\left( {{\text{r }}0.{8452}} \right)$$

### Fixed time method

At specific times, the absorption of the solutions containing varying amounts of CLN against a blank solution was measured.

Graphically. The curve was built by representing absorption values versus the concentration of (CLN.HCl) at a preselected time of 0–2400 s (40 min).

The correlation coefficient, slope and intersection value were determined at each time, and these values are shown in Table 1. The most acceptable value and which had the best R-value was obtained.

**Fig. 7:**
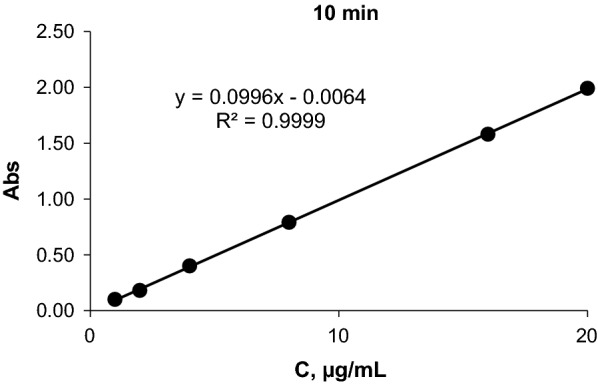
Graph for determining CLN.HCl by fixed time method (t = 10 min)

Consequently, a fixed time of 600 s was selected to determine (CLN.HCl). (Fig. 7).

### Evaluate the validity of the proposed method

#### Calibration graph

The Correlation coefficient, intercept and slope values for the calibration data were determined by using the least square method [20]. The most appropriate values of the correlation coefficient were found for the reaction at 600 s as a fixed time, which was chosen as the best and most efficient time for doing the measurements and determine the drug (Table 2).

Reaction conditions were stated, then a fixed time method was used to estimate CLN.HCl in a range of concentration of 2–12 mg / L. Limit of determination that can be detected (LOD) was found by the proposed method, the limit of quantification was also found (LOQ) (Table2).

#### Accuracy and precision

The accuracy and precision of the method were confirmed by performing five measurements of different drug concentrations. The value of the recovery and relative standard deviation were good and within the acceptable limits. That demonstrated that the method was of good efficacy. The results are presented in Table 3.

**Table 3:** Accuracy and precision for the determination of CLN.HCL in bulk powder by the proposed method:

Drug	Method	mg/ml			RSD %	%Recovery
		Taken	Found	S.D*		
*CLN.HCl*	*Fixed time*	1	1.018	0.011	1.08	101.81
		2	1.996	0.034	1.68	99.8
		4	3.98	0.013	0.33	99.5
		8	8.056	0.022	0.27	100.7
		16	16.069	0.075	0.47	100.43
		20	20.337	0.398	1.96	101.69
	*Initial rate*	1	1.02	0.017	1.67	102
		2	2.035	0.04	1.97	101.75
		4	3.93	0.012	0.31	98.25
		8	8.1	0.12	1.48	101.25
		16	16.1	0.2	1.24	100.63
		20	19.89	0.32	1.61	99.45

#### Analysis of Clindamycin Hydrochloride in capsules

To estimate CLN.HCl in capsules, an advanced kinetic spectrophotometric method was used to identify Clindamycin in drug samples, and the results were summarized in Table 4.

**Table 4:** Estimation of CLN.HCl in their pharmaceutical preparations using the proposed kinetic methods and reference methods:

Formula	Drug	Claim (mg/tab)	Recovery % ± S.D*		Reference method [3]
			Proposed kinetic method		
			Fixed time	Initial rate	
Clindo**	CLN	75	99.13% ± 2.07 t = 1.76 F = 2.83	98. 97 ± 1.34 t = 1.64 F = 2.87	100.16% ± 0.25 t = 1.42
		150	101.12% ± 0.63 t = 2.08 F = 2.45	100.83% ± 1.36 t = 1.93 F = 2.39	101.16% ± 1.02 t = 1.54
		300	100.18% ± 0.99 t = 1.84 F = 2. 13	100.89% ± 1.03 t = 2.45 F = 1.98	100.75% ± 0.28 t = 1.13
			Fixed time	Initial rate	
Clindamycin Biomed***	CLN	75	99.19% ± 0.69 t = 0.16 F = 2.39	100.15% ± 1.38 t = 1.56 F = 2.18	100.53% ± 1.02 t = 0.83
		150	99.25% ± 1.17 t = 1.46 F = 2.37	100.25% ± 0.87 t = 2.10 F = 2.67	101.19% ± 1.00 t = 0.79
		300	100.29% ± 1.19 t = 1.18 F = 2.72	101.50% ± 1.05 t = 1.82 F = 2.28	100.76% ± 0.89 t = 1.09

At 95% confidence limit, t- and F value at five degrees of freedom are t = 2.776 and f = 6.26

*Recovery is the mean of five replicates

**Product Supplied by AL-SAAD pharmaceutical industries, Syria

***Product Supplied by BIOMED pharmaceutical industries, Syria

The proposed kinetic spectrophotometric method has the advantage of not interfering with excipients in pharmaceutical forms. The results of F and T-tests were shown in Table 4.

When the results obtained using the proposed method were compared with those obtained when applying the reference method[3]. The results were within acceptable limits, indicating that the proposed method is accurate because there is no significant difference between the proposed method and the reference method.

## Conclusion

The proposed method is easy, fast, inexpensive, environmentally friendly and does not use any organic materials or reagents.

It is also having good recovery, accuracy and precision. The method can be used in the routine determination of clindamycin as a raw material or capsules.

The results obtained indicate that there is no interference with the excipients.

the proposed method is the first kinetic spectrophotometric study used to determine the concentration of clindamycin hydrochloride. All analytical reagents are reasonably priced, and the present method is environmentally safe because it doesn’t need any organic reagents or solvents, it is free extractive and also very sensitive comparing with the other spectrophotometric methods.

## Data Availability

The article includes the sources of all materials which was used in the research. The data are available from the corresponding author on reasonable request.
